# Hemodynamic response and clinical outcome following intravenous milrinone plus norepinephrine–based hyperdynamic hypertensive therapy in patients suffering secondary cerebral ischemia after aneurysmal subarachnoid hemorrhage

**DOI:** 10.1007/s00701-022-05145-6

**Published:** 2022-02-09

**Authors:** Hans-Jakob Steiger, Rolf Ensner, Lukas Andereggen, Luca Remonda, Jatta Berberat, Serge Marbacher

**Affiliations:** 1 Department of Neurosurgery, Neurozentrum, Kantonsspital Aarau, Aarau, Switzerland; 2 Klinik Für Neurochirurgie, Neurozentrum, Kantonsspital Aarau, Tellstr. 25, CH-5001 Aarau, Switzerland; 3 Surgical Intensive Care Unit, Kantonsspital Aarau, Aarau, Switzerland; 4 Division of Neuroradiology, Kantonsspital Aarau, Aarau, Switzerland

**Keywords:** Milrinone, Subarachnoid hemorrhage, Delayed cerebral ischemia, Vasospasm, Intra-arterial spasmolysis, Nimodipine

## Abstract

**Purpose:**

Intravenous and intra-arterial milrinone as a rescue measure for delayed cerebral ischemia (DCI) after subarachnoid hemorrhage (SAH) has been adopted by several groups, but so far, evidence for the clinical benefit is unclear and effect on brain perfusion is unknown. The aim of the actual analysis was to define cerebral hemodynamic effects and outcome of intravenous milrinone plus norepinephrine supplemented by intra-arterial nimodipine as a rescue strategy for DCI following aneurysmal SAH.

**Methods:**

Of 176 patients with aneurysmal SAH treated at our neurosurgical department between April 2016 and March 2021, 98 suffered from DCI and were submitted to rescue therapy. For the current analysis, characteristics of these patients and clinical response to rescue therapy were correlated with hemodynamic parameters, as assessed by CT angiography (CTA) and perfusion CT. Time to peak (TTP) delay in the ischemic focus and the volume with a TTP delay of more than 4 s (T4 volume) were used as hemodynamic parameters.

**Results:**

The median delay to neurological deterioration following SAH was 5 days. Perfusion CT at that time showed median T4 volumes of 40 cc and mean focal TTP delays of 2.5 ± 2.1 s in these patients. Following rescue therapy, median T4 volume decreased to 10 cc and mean focal TTP delay to 1.7 ± 1.9 s. Seventeen patients (17% of patients with DCI) underwent additional intra-arterial spasmolysis using nimodipine. Visible resolution of macroscopic vasospasm on CTA was observed in 43% patients with DCI and verified vasospasm on CTA, including those managed with additional intra-arterial spasmolysis. Initial WFNS grade, occurrence of secondary infarction, ischemic volumes and TTP delays at the time of decline, the time to clinical decline, and the necessity for additional intra-arterial spasmolysis were identified as the most important features determining neurological outcome at 6 months.

**Conclusion:**

The current analysis shows that cerebral perfusion in the setting of secondary cerebral ischemia following SAH is measurably improved by milrinone and norepinephrine–based hyperdynamic therapy. A long-term clinical benefit by the addition of milrinone appears likely. Separation of the direct effect of milrinone from the effect of induced hypertension is not possible based on the present dataset.

## Introduction

Hypertensive normo- or hypervolemic treatment has been the backbone for managing delayed cerebral ischemia (DCI) following aneurysmal subarachnoid hemorrhage (SAH) for almost half a century [9, 22, 24, 29]. Intra-arterial and intravenous milrinone has become popular over the last two decades and a number of clinical series have been published. Milrinone as an inotropic vasodilator promises augmentation of perfusion as well as resolution of the arterial constriction [12]. Reports regarding the clinical effect in patients with DCI and vasospasm after SAH paint a heterogeneous picture so far [1, 3, 6–8, 12, 28, 31]. An immediate clinical benefit is described in the majority of the reports and intra-arterial application appears to have a clear antispastic effect with visible vascular relaxation. In contrast, the impact on long-term outcome and effect of intravenous application on vascular relaxation and cerebral perfusion in patients with SAH are not well known.

We adopted at our hospital intravenous milrinone plus norepinephrine–based rescue therapy in 2016 as the standard for managing symptomatic cerebral vasospasm respectively DCI following SAH. Since milrinone can lead to arterial hypotension or at least counteracts blood pressure and arterial pressure levels recommended by current international guidelines [9], rescue therapy required the additional use of vasopressors. The treatment protocol included a strict schedule of perfusion CT examinations to monitor cerebral perfusion. The flowchart also included the option of additional intra-arterial nimodipine infusion in case of insufficient effect of systemic therapy. The purpose of the actual analysis was to define the effect on brain perfusion and to quantify the clinical benefit of our rescue strategy.

## Methods

**Fig. 1:**
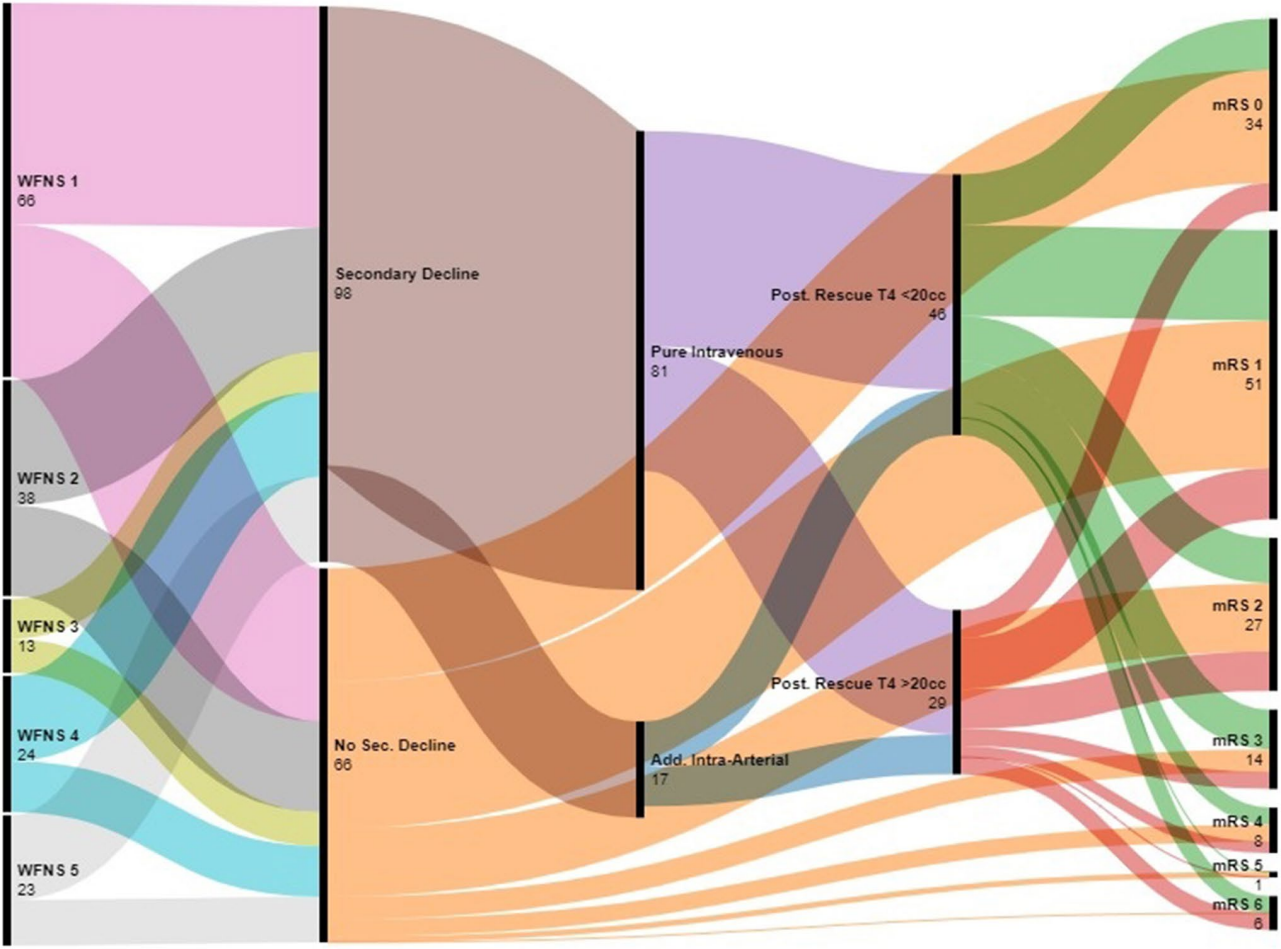
Flowchart of the entire group of patients, comparing patients with secondary deterioration and patients without. Twelve patients in extremely poor condition who died early were excluded from this comparison. Overall, patients suffering secondary decline had a somewhat less favorable outcome at 6 months compared to patients not suffering secondary neurological decline (*P* = 0.008). The numbers correspond to the patients in the respective subgroups with the pertinent data available

Rescue therapy for secondary ischemic decline following aneurysmal SAH has been used at our department in a consistent manner since January 2016. The current retrospective review of the hemodynamic results and the clinical outcome includes patients treated between April 2016 and March 2021. Of 176 patients with aneurysmal SAH managed at the interdisciplinary neuroscience center between April 2016 and March 2021, 98 (56%) suffered from secondary ischemic deterioration and were submitted to milrinone-based rescue therapy (see Fig. 1). Patient charts and imaging were reviewed for the actual analysis. Patient characteristics and outcome were correlated with hemodynamic and clinical response to rescue therapy.

### Clinical treatment protocol

Care and active treatment of our patients with aneurysmal SAH followed current international guidelines [9]. Initial diagnosis of SAH was achieved with CT and CTA, and if inconclusive supplemented by MRI and/or spinal tap. Patients with decreased level of consciousness (WFNS grades 4 and 5) were managed with an external ventricular drainage, intubation, and controlled ventilation at least until the aneurysm was obliterated by endovascular or surgical route. Systolic blood pressure was maintained below 140 mmHg prior to aneurysm obliteration. Possible anticoagulants were stopped upon admission and antagonized if possible.

Aneurysm diagnosis was completed by two- and three-dimensional digital subtraction angiography within 24 h after admission. The mode of aneurysm occlusion was chosen by interdisciplinary discussion.

Following surgical or endovascular aneurysm occlusion, normal blood pressure and blood volume were aimed at, but preventive hypertensive management was not used. No spasmolytic such as nimodipine or fasudil was used prophylactically. Thromboprophylaxis using fractionated heparin (nadroparin) was given from day 1 following aneurysm occlusion except when regular heparin was administered for specific problems following the endovascular or surgical procedure.

Baseline CTA and perfusion CT was done on the day after aneurysm occlusion and then again on days 5 and 10 after intervention. If new neurological deficits occurred during the course, CTA and perfusion CT were repeated immediately. In case of impaired perfusion, hyperdynamic management was begun following exclusion of space-occupying hematoma, swelling, electrolyte imbalance, or epileptic activity [30].

Hyperdynamic management included the use of intravenous milrinone at a dose of 0.75–2.5 μg/kg/min and induced hypertension to a mean arterial pressure of 90–120 mmHg using norepinephrine, and normovolemia. Milrinone dosing was oriented according to the recommendations for congestive heart failure. Since elimination of the drug occurs mainly via the renal pathway, adaptions of dosing were made in case of renal impairment. Relatively frequent side effects of milrinone (3–10%) are cardiac arrhythmias, headache, and hypotension. In order to maintain desired blood pressure levels in the case of symptomatic vasospasm, almost all patients needed norepinephrine blood pressure support.

Following initiation, milrinone and norepinephrine were maintained for at least 48–72 h, before trying to wean again as based on neurological condition and perfusion on CT. If symptoms recurred following reduction, the dose was increased again.

CTA and perfusion CT were repeated within 24 to 48 h after initiation of rescue therapy, depending on the clinical response. In case of insufficient response, persistent ischemic symptoms, and perfusion delay on perfusion CT, intra-arterial slow infusion of nimodipine was chosen as a last resort (2–4 mg, corresponding to 12 ml in 38 ml saline, administered at 20–30 ml per hour for 30 min).

### Data extraction and analysis

Data extracted from the patients’ records included demographic, clinical, and radiological parameters. Diagnosis of macrospasm was based on segmental reduction of the vascular diameter by 50% or more in the ICA, BA, M1, M2, A1, A2, P1, or P2 arterial segments. Perfusion CT analysis was performed with the Vitrea™ package (Vital Europe, Veenendaal, The Netherlands) and the RAPID™ summary tool (RapidAI, Menlo Park, CA, USA). The volume with a TTP delay of more than 4 s (T4 volume) and the TTP delay of the ischemic core compared to a reference region of interest (ROI) were chosen as parameters to quantify the extent of ischemia. In case of MCA territory ischemia, reference ROI was placed symmetrically in the contralateral hemisphere. In case of ACA or PCA ischemia, reference ROI was positioned in the MCA territory of the less affected hemisphere.

Clinical and hemodynamic parameters were compared before and after induction of rescue therapy. The variables were also compared between the group with effective reversal of secondary ischemia resulting in a T4 volume under 20 cc, and less effective therapy, respectively, and also between the patients enjoying a favorable recovery (mRS at 6 months 0–1) and the ones with a less favorable outcome (mRS ≥ 2).

Data were analyzed using IBM SPSS statistical software version 24.0 (IBM Corp., New York, NY, USA). Continuous variables were examined for homogeneity of variance and are expressed as mean ± SD unless otherwise noted. Categorical variables are given as numbers and percentages. For comparison of means between groups, Student’s *t*-test was used for normally distributed data, and the Mann–Whitney test for nonparametric data. Categorical variables were compared using Pearson’s chi-square test or Fisher’s exact test, as appropriate. Odds ratios (ORs) and 95% confidence intervals (CIs) of independent hemodynamic and clinical parameters related to unfavorable outcome (mRS ≥ 2) at 6 months in patients with secondary decline were analyzed by univariate and multivariate binary logistic regression model. The multivariable logistic regression analysis included all dependent risk factors in the univariable regression with a *P* value ≤ 0.05. Significance level was set at 5%. Graphical work was done using the Python package (https://www.python.org), the included MatPlotlib module, the scikit-learn platform (https://scikit-learn.org/stable/), and the RAWgraphs 2.0 graphic tool (https://app.rawgraphs.io/).

## Results

The entire cohort including patients without secondary decline consisted of 66, 39, 13, 26, and 32 admissions in WFNS grades 1, 2, 3, 4, and 5, respectively. Seventy-seven patients underwent endovascular aneurysm obliteration and 90 microsurgical treatment. The aneurysm was left untreated in 9 cases due to poor clinical condition. At 6 months, 40, 57, 29, 16, 10, 1, and 22 had recovered to mRS 0, 1, 2, 3, 4, 5, and 6, respectively. One patient was lost for follow-up.

Ninety-eight patients suffered secondary ischemic symptoms or neurological deficits following aneurysm occlusion (Fig. 1). These patients did not differ significantly from patients without secondary deterioration regarding median age (58 years, *P* = 0.54), median initial WFNS grade (2, *P* = 0.35), and Fisher grade (4, *P* = 0.84). However, patients suffering secondary decline had already a higher ischemic T4 volume on the initial post interventional baseline perfusion CT (median T4 volume 8.0 versus 0 cc, *P* = 0.04). Overall, patients suffering secondary decline had a less favorable outcome at 6 months with a median mRS of 1 (IQR 1–3) compared to patients not suffering secondary neurological decline with a median mRS of 1 (IQR 0–2, *P* = 0.008). Twelve patients in extremely poor condition who died during the first days were excluded in this comparison.

Regarding the patients suffering of secondary neurological decline, the median delay to neurological deterioration following SAH was 5 days (IQR 2.25–7). Perfusion CT at that time showed median T4 volumes of 40 cc (IQR 20–87.5) and an average focal TTP delay of 2.5 ± 2.1 s in these patients. Segmental narrowing of major cerebral arteries to less than 50% diameter was seen on CTA in 69 of 92 of the patients suffering secondary decline and with adequate CTA at the time of decline (75%).

**Fig. 2:**
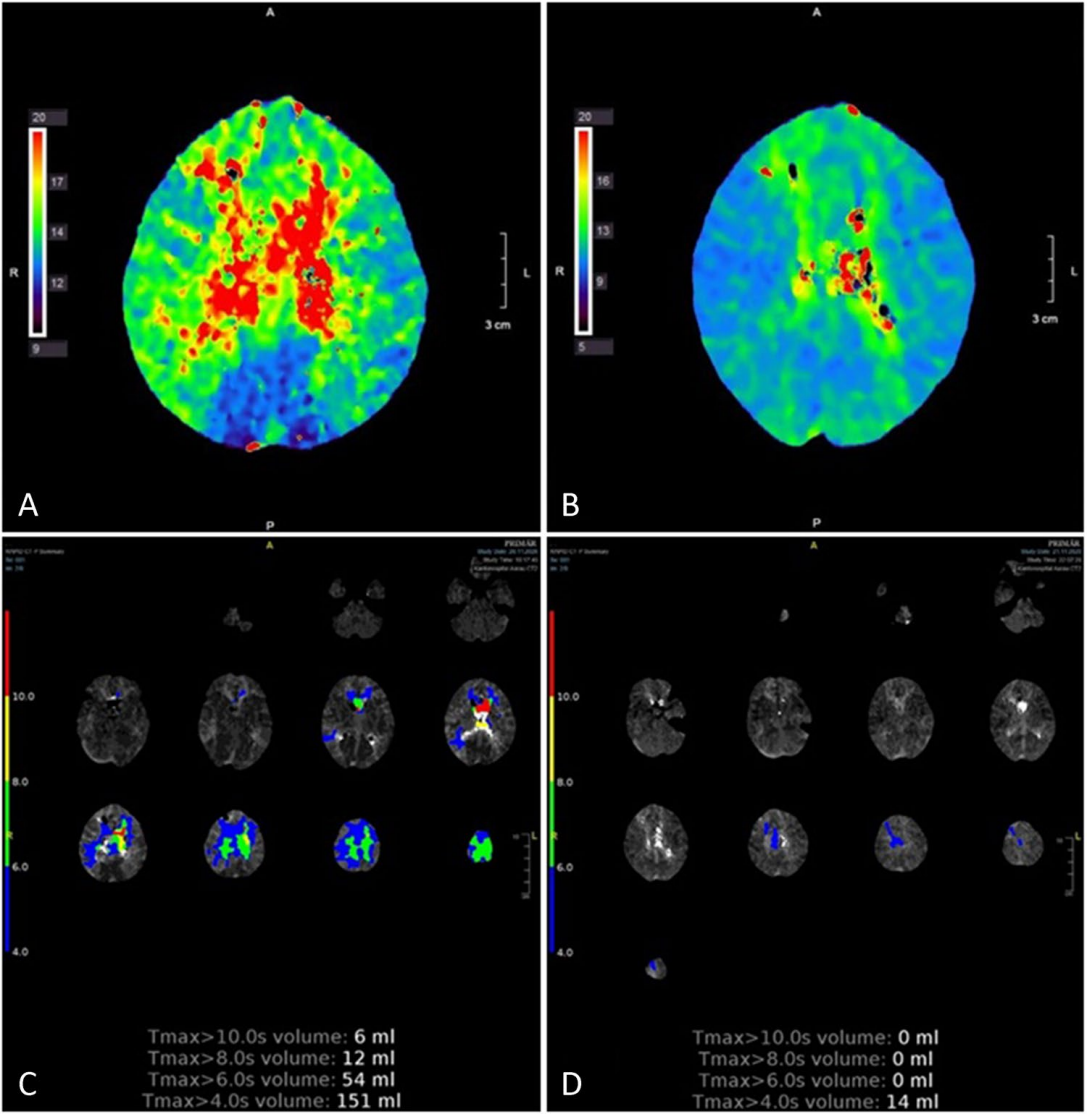
Example of TTP map (upper row, A and B) and T4 volume (lower row, C and D) at the time of decline 5 days following SAH from an anterior communicating artery aneurysm and surgical clipping, showing delayed TTP mainly in the anterior cerebral artery territories and a T4 volume of 151 cc (left, A and C). Control exam 1 day later after induction of hyperdynamic rescue therapy (right, B and D). Following rescue therapy, TTP was clearly shortened in the previously delayed anterior cerebral artery territory but also in the other regions. Rescue therapy reduced median T4 volume to 14 cc

Following institution of rescue therapy, median T4 volume was reduced to 10 cc (IQR 4–30) and average focal TTP delay to 1.7 ± 1.9 s (see Figs. 1 and 2, Table 1). A total of 17 of the 98 patients (17%) underwent additional intra-arterial nimodipine spasmolysis due to insufficient clinical response and improvement of perfusion as measured by CTP. Segmental macrospasm was improved to less than 50% stenosis in 26 of the 61 patients with macroscopic vasospasm and appropriate follow-up CTA, including 9 undergoing additional intra-arterial nimodipine spasmolysis (43%). Excluding those 9 patients would result in a rate of resolution of macrospasm under pure intravenous hyperdynamic therapy of 33%.

**Table 1:** Patients with secondary cerebral ischemia—hemodynamic key values at the time of secondary decline and after institution of rescue therapy

Parameter	Day of decline	Post rescue therapy
T4 volume (median, IQR)	40 (20, 88)	10 (4, 30)
TTP ischemic zone (mean ± STD)	15.8 s ± 3.6 s	13.4 ± 3.3 s
TTP reference (mean ± STD)	13.3 s ± 3.0 s	11.7 ± 2.6 s
TTP difference (mean ± STD)	2.5 ± 2.1 s	1.7 ± 1.9 s

Parameters related to successful (T4 volume < 20 cc) and less successful rescue therapy are compared in Table 2. Baseline ischemic volumes and the occurrence of secondary infarction at the time of discharge differed significantly between these groups. Median outcome mRS at 6 months was 1 (IQR 1–3) in the group with effective rescue therapy versus 2 (IQR also 1–3) in the patients with less effective therapy, a difference which was not statistically significant (*P* = 0.63).

**Table 2:** Patients with secondary cerebral ischemia—parameters related to successful and unsuccessful rescue therapy in terms of post rescue ischemic volume (volume with TTP delay > 4 s, T4 volume)

	T4 volume < 20 cc	T4 volume ≥ 20 cc	*P*-value
Age (median, IQR)	61 (54, 68)	56 (46, 62)	0.07
Gender (% female)	79%	61%	0.13
Diabetes (%)	7%	0%	0.28
Hypertension (%)	50%	38%	0.35
WFNS grade (median, IQR)	2(1, 4)	2 (1, 4)	0.52
Fisher grade (median, IQR)	4(3, 4)	4 (2, 4)	0.40
Posterior circulation aneurysm (%)	11%	7%	0.70
Aneurysm size (mm, median, IQR)	7(5, 10)	7 (5, 14)	0.26
Aneurysm occlusion (% coiled)	44%	32%	0.46
Post occlusion WFNS (median, IQR)	2 (2, 4)	2 (1, 4)	0.86
Post occlusion T4 volume (median, IQR)	9 (0, 30)	10 (5, 58)	0.07
Day of decline (median, IQR)	5 (2, 7)	6 (3, 8)	0.49
T4 volume on day of decline (median, IQR)	28 (10, 60)	60 (40, 100)	0.03
Focal TTP delay on day of decline (mean ± STD)	2.1 ± 1.5 s	2.9 ± 2.1 s	0.05
Macrospasm on CTA (%)	64%	93%	0.01
Additional intra-arterial spasmolysis (%)	18%	25%	0.56
Post rescue T4 volume (median, IQR)	5 (0, 10)	30(23, 50)	0.001
Post rescue focal TTP delay (mean ± STD)	1.3 ± 1.5 s	2.4 ± 2.3 s	0.01
Resolution of macrospasm (%)	42%	44%	1.0
Secondary infarction (%)	11%	32%	0.03
mRS at 6 months (median, IQR)	1 (1, 3)	2 (1, 3)	0.63

**Fig. 3:**
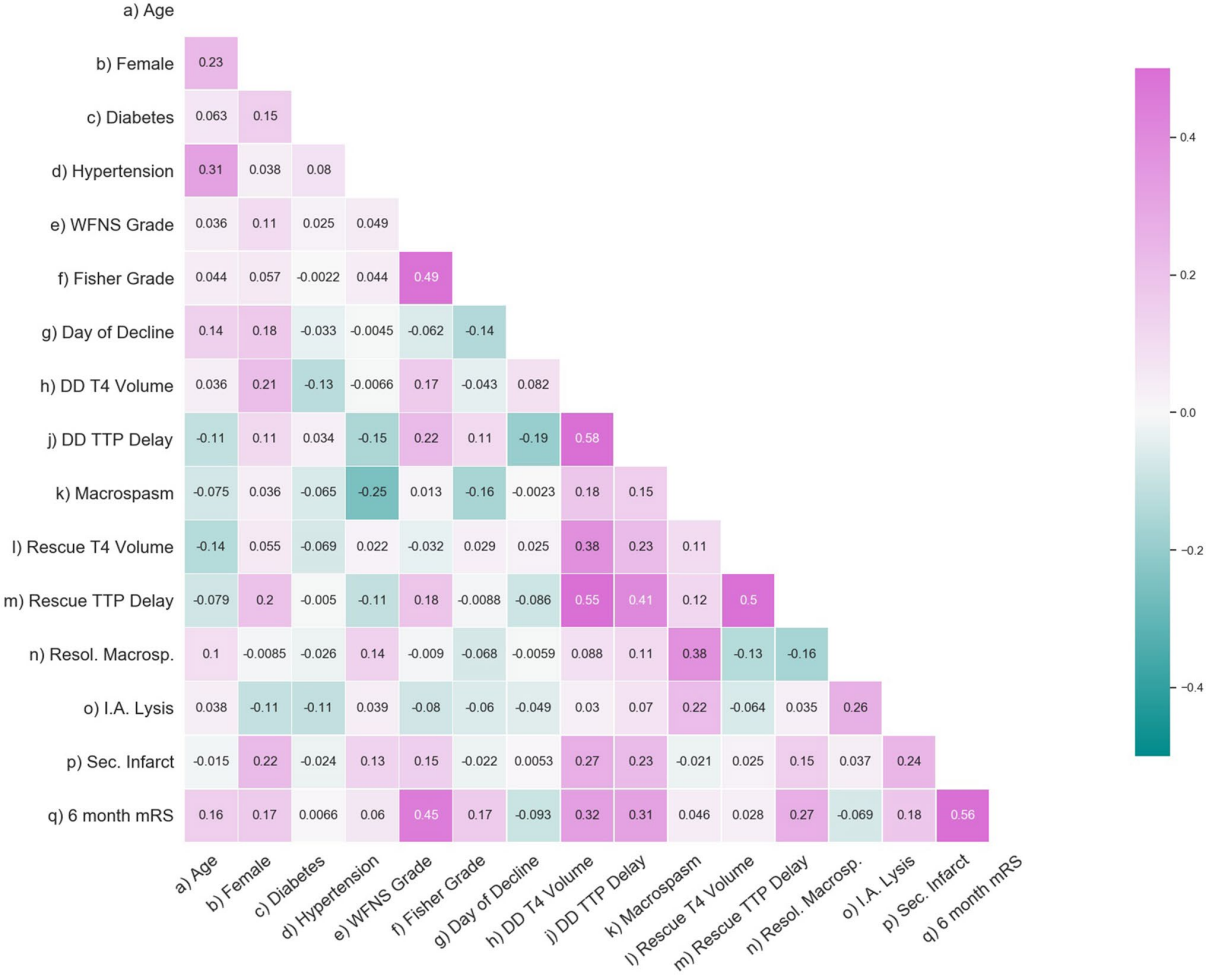
Pearson correlation matrix showing interactions between clinical and hemodynamic factors possibly involved in the result of rescue intervention. The analysis correlates, apart from trivial interactions, mainly visible secondary infarcts at the time of discharge, higher WFNS grade, and larger ischemic volumes with an unfavorable 6-month mRS. Necessity for additional intra-arterial spasmolysis, higher age and female gender also correlated modestly with 6-month mRS

Pearson correlation matrix and univariate comparison associated mainly visible secondary infarcts at the time of discharge, higher WFNS grade, larger ischemic volumes, and early occurrence of secondary deterioration with an unfavorable 6-month mRS (Table 3, Fig. 3). Further non-trivial correlations showed that secondary infarcts at the time of discharge correlated moderately with T4 volume and TTP delay at the time of decline, and with the necessity for intra-arterial spasmolysis. Resolution of macrospasm correlated moderately with intra-arterial spasmolysis.

**Table 3:** Patients with secondary cerebral ischemia—parameters related to favorable (mRS 0, 1) and unfavorable (mRS ≥ 2) clinical outcome at 6 months

	mRS 0–1	mRS ≥ 2	P-value
Age (median, IQR)	57 (50, 64)	61 (52, 66)	0.20
Gender (% female)	67%	72%	0.66
Diabetes (%)	4%	6%	0.67
Hypertension (%)	39%	47%	0.54
WFNS grade (median, IQR)	1 (1, 2)	4 (2, 5)	< 0.00001
Fisher grade (median, IQR)	3 (3, 4)	4 (4, 4)	0.02
Posterior circulation aneurysm (%)	5%	3%	0.71
Aneurysm size (mm, median, IQR)	6 (5, 9)	6 (5, 10)	0.17
Aneurysm occlusion (% coiled)	44%	39%	1
Post occlusion WFNS (median, IQR)	2 (1, 2)	4 (3, 5)	< 0.000001
Post occlusion T4 volume (median, IQR)	2.5 (0, 16)	10 (5, 50)	0.03
Day of decline (median, IQR)	6 (4, 8)	5(2, 7)	0.01
T4 volume on day of decline (median, IQR)	36 (10, 72)	50 (28, 99)	0.08
Focal TTP delay on day of decline (mean ± STD)	2.0 ± 1.5 s	3.1 ± 2.6 s	0.02
Macrospasm on CTA (%)	69%	81%	0.23
Additional intra-arterial spasmolysis (%)	10%	29%	0.03
Post rescue T4 volume (median, IQR)	10 (0, 25)	15 (5, 30)	0.93
Post rescue focal TTP delay (mean ± STD)	1.3 ± 1.2 s	2.1 ± 2.4 s	0.08
Resolution of macrospasm (%)	41%	45%	0.80
Secondary infarction (%)	10%	36%	0.003

In the multivariate analysis, WFNS grade (*P* < 0.001) and the occurrence of secondary infarcts (*P* = 0.03) remained the most significant prognostic factors for the outcome at 6 months.

## Discussion

Prevention and management of symptomatic vasospasm have undoubtedly made great progress over the past decades, although the scientific evidence, particularly for preventive measures, remains relatively thin. Only oral nimodipine and intravenous fasudil could be convincingly shown to reduce the rate of symptomatic vasospasm and poor outcome in randomized trials [2, 27]. Later in smaller prospective trials, cilostazol, like milrinone, a selective phosphodiesterase 3-inhibitor, also showed promising results [20, 26]. Regarding management of symptomatic vasospasm, the most convincing argument for a specific strategy often does not come from randomized trials focusing on long-term outcome, but rather from the immediately visible effect on the neurological deficits. Immediate improvement of symptoms and neurological deficits after institution of rescue therapy leaves little doubt regarding efficacy. At our institution, the often strikingly visible clinical improvement was also the reason to accept intravenous milrinone as the standard rescue therapy following initial use as a measure of last resort. Clinical improvement was judged based on focal neurological symptoms, level of consciousness, and headache. The high degree of suspicion may well have led to initiation of rescue therapy in some cases who would also have recovered with less. On the other hand, the good tolerability of milrinone in the light of the severe and irreversible consequences of occurring infarction justified a relatively generous indication in our view.

We did not use oral nimodipine prophylactically. At our institution, we had a larger proportion of poor grade patients compared to the Allen study, which would have required intravenous application, which is not recommended [1, 9].

We based assessment of brain perfusion on perfusion CT. This method provided in our experience reasonable results. The currently available RapidAI and Vitrea software helped interpretation and reproducibility. Interaction of coil and clip artifacts with the measurements, particularly the arterial reference at the base of skull, cannot be a 100% excluded. However, both the automatic RapidAI software and the manual definition of the arterial reference check for a proper density dynamic of the selected pixels.

We used routine baseline CTA and perfusion CT on the day after aneurysm occlusion and then again on days 5 and 10 after intervention. The question obviously arises, how often the routine imaging at 5 or 10 days triggered the treatment protocol if patient was asymptomatic, or what was the need for routine imaging in asymptomatic patients. Usually indication for initiating hyperdynamic treatment was a combination of clinical symptoms and imaging data. In patients with depressed level of consciousness, imaging data became more important. Almost half of our patients undergoing rescue therapy had a severely depressed level of consciousness at the time of initiation of rescue therapy (GCS < 12). Most of them were initially admitted in WFNS 4 or 5.

Forty years ago, HHH (hypervolemia, hypertension, hemodilution) therapy became the mainstay of treatment for vasospasm following subarachnoid hemorrhage [24, 29]. Volume expansion using crystalloid and colloid solutions was subsequently discredited in a number of settings and further analysis for the case of subarachnoid hemorrhage indicated that hemodilution and plasma expansion added little to the effect of induced hypertension, or were even contra productive [4, 22]. Milrinone became popular as an inotropic agent because of the associated vasodilation effects [12]. While improvement of angiographic vasospasm could be shown after intra-arterial application, the effect following intravenous application remains unclear [3, 10]. A few case reports and a small comparative study demonstrated decreasing flow velocities after intravenous milrinone therapy as measured with transcranial Doppler [11, 23, 25]. Clarifying the effect of intravenous milrinone on cerebral perfusion and macroscopic vasospasm was the main aim of the current analysis. Since additional norepinephrine was used to maintain the desired level of arterial pressure, our analysis can obviously not differentiate between the effects of milrinone and norepinephrine.

The clinical outcome of our entire group of patients suffering aneurysmal SAH did not substantially differ from other recent series, with and overall rate of approximately one third of patients recovering poorly to a mRS > 2 [15, 18, 21]. The overall rate of delayed cerebral ischemia in our series appears rather high compared with other recent series, suggesting high degree of suspicion and low threshold for the initiation of rescue therapy at our center [13, 16].

Overall, although our patients suffering secondary decline still had a somewhat less favorable outcome at 6 months compared to patients without secondary neurological decline, the difference was relatively modest (median mRS 1, IQR 1–3 compared to median mRS 1, IQR 0–2). We must assume that without the consequent rescue therapy, the difference would have been more pronounced. Lacking a control group, however, our data cannot prove or disprove this suspicion. So far, few studies have compared intravenous milrinone with other rescue therapies for vasospasm and DCI (see Table 4). Labeyrie and coworkers compared the strategies of intensive therapy for delayed cerebral ischemia after subarachnoid hemorrhage of two hospitals, one of them using as similar milrinone-based management as in the current series, and the other using only induced hypertension [13]. These authors did not see a substantial difference in outcome between the two strategies. In contrast, Lakhal et al. reported from their monocentric historical comparison significantly lower rates of brain infarction and poor clinical outcome using intravenous milrinone in combination with induced hypertension, compared to induced hypertension alone [14].

**Table 4:** Comparative clinical studies using intravenous milrinone for vasospasm and DCI following aneurysmal subarachnoid hemorrhage

Author, year	Type of study	Total number of patients	Additional measures in milrinone group	Measures in control group	Primary outcome	Result
Soliman, 2019 [28]	Prospective, preventive, randomized	90	None, i.v. milrinone for 21 days	Intravenous magnesium for 21 days	TCD flow velocity < 120 cm/s, DCI	Magnesium better in terms of TCD and occurrence of DCI
Rouanet, 2021 [23]	Prospective, observational	21	None	Norepinephrine	TCD values and NIHSS at 45 and 90 min	TCD value lower in milrinone group, no clinical difference
Labeyrie, 2021 [13]	Retrospective two site comparison	200	Balloon angioplasty	Induced hypertension	1-month mortality, 6-month mRS 0–2, brain infarction	No or minimal difference between strategies
Lakhal, 2021 [14]	Retrospective historical comparison	94	Induced hypertension	Hypertension alone	6-month mRS 2–6, brain infarction	mRS 2–6 and infarction 75% lower in treatment group

The main predictors determining the outcome in our patients suffering secondary decline remained the known factors determining the prognosis with aneurysmal SAH in general, i.e., initial WFNS grade. In addition, T4 volume on the day of decline and corresponding TTP delay, and the occurrence of secondary infarction by the time of discharge were found significant for the prognosis at 6 months. Furthermore, patients experiencing a secondary decline early fared somewhat worse than patients with a later onset did. In a recent secondary analysis of several trial cohorts, Martini and coworkers identified younger age and absence of cerebral ischemia/infarction as the principal factors determining a good outcome at 3 months in patients suffering secondary decline [19]. Age was of minor importance in our univariate comparison.

Regarding the cerebral hemodynamic effect of hyperdynamic rescue therapy, our CT perfusion studies showed that the median T4 volumes of 40 cc at the time of decline could be reduced to 10 cc with the hyperdynamic therapy, and the average focal TTP delay of 2.5 s to 1.7 s. While several reports describe a direct effect on vasospasm of larger arteries following intra-arterial and intra-cisternal application of milrinone, reports on cerebral perfusion and vasospasm after i.v. application are scarce [3, 25]. Zeiler and colleagues described a case of angiographic resolution of vasospasm with intravenous milrinone [31]. We saw visible reduction of macroscopic vasospasm on CTA more frequently but not exclusively in our patients undergoing additional intra-arterial spasmolysis using nimodipine.

In general, clinical response and improvement of perfusion on CT were in line. Nonetheless, there was one patient (WFNS 5) in whom rescue therapy was started with already visible infarctions in the ACA territory. T4 volume was 331 cc at the time and was reduced to 19 cc following institution of rescue therapy with additional intra-arterial spasmolysis. Despite improved perfusion, he subsequently developed intracranial pressures up to 80 mmHg. No surgical decompression was wished by the family and the patient died. Although expanding infarction is the most likely course of intracranial hypertension, a contributing effect of milrinone or intra-arterial nimodipine cannot be excluded entirely. Otherwise, we did not see serious complications in the current series related to the use of milrinone and induced hypertension. Hypertensive therapy based on vasopressors alone is not without risk and particularly splanchnic ischemia may be a concern [17]. In this series, we did not observe such complications. It is possible that milrinone compares favorably with epinephrine-based regimens, but so far, we do not know.

Decision for reduction was based on neurological condition and perfusion on CT. If symptoms recurred following reduction, the dose was increased again. We did not correlate dose and clinical and perfusion effect respectively. This would also require correction for body weight and renal function.

As mentioned, our protocol for refractory vasospasm with persistent clinical symptoms and radiographic hypoperfusion included intra-arterial infusion of nimodipine. The outcome at 6 months in these patients was clearly less favorable compared the patients who could be managed with intravenous milrinone alone. The worse prognosis is obviously due to the more severe condition of the patients undergoing additional intra-arterial spasmolysis. Perfusion CT indicated that treatment resulted in a similar distribution regarding post rescue T4 volume as for patients without the need for additional intra-arterial nimodipine. Furthermore, correlation analysis showed a moderate connection between visible improvement of macrospasm and intra-arterial spasmolysis.

### Strengths and limitations

The main strength of the current analysis is the perfusion CT quantification of the immediate cerebral hemodynamic effect of the institution of milrinone and norepinephrine–based rescue therapy, which to our knowledge has not been quantified before. The strongest evidence from our analysis is the comparison of the perfusion CTs before and after induction of the milrinone-based rescue therapy. Nonetheless, the currently available methods for quantifying perfusion CT parameters are not perfect and the definition of the ischemic and reference ROIs was subject to some judgment. Formally, the analysis corresponded to the analysis of an interrupted time series, as commonly used in epidemiology to quantify public health interventions [5]. The trend after an intervention is compared to the time before. The challenge here is the definition of the counterfactual model, i.e., what would have happened without the intervention. We assume that after diagnosis of vasospasm and secondary ischemia, symptoms and perfusion parameters would have got worse without the intervention, and that the long-term outcome of the patients suffering secondary neurological deficits would have been less favorable. Since this was a retrospective analysis without control group undergoing a more traditional rescue management, the hypothesis cannot be formally proven.

## Conclusions

In conclusion, the current analysis shows that cerebral perfusion in the setting of secondary cerebral ischemia following SAH is measurably improved by milrinone and norepinephrine–based hyperdynamic hypertensive therapy supplemented by intra-arterial nimodipine spasmolysis if necessary. A long-term clinical benefit appears likely, although lacking a control group, formal proof cannot be offered. Separation of the direct effect of milrinone from the effect of induced hypertension is not possible based on the present dataset.
